# Intelligent Millimeter-Wave System for Human Activity Monitoring for Telemedicine

**DOI:** 10.3390/s24010268

**Published:** 2024-01-02

**Authors:** Abdullah K. Alhazmi, Mubarak A. Alanazi, Awwad H. Alshehry, Saleh M. Alshahry, Jennifer Jaszek, Cameron Djukic, Anna Brown, Kurt Jackson, Vamsy P. Chodavarapu

**Affiliations:** 1 Department of Electrical and Computer Engineering, University of Dayton, 300 College Park, Dayton, OH 45469, USA; alhazmia3@udayton.edu (A.K.A.); alshehrya1@udayton.edu (A.H.A.); alshahrys1@udayton.edu (S.M.A.); 2 Electrical Engineering Department, Jubail Industrial College, Royal Commission for Jubail and Yanbu, Jubail Industrial City 31961, Saudi Arabia; alanazi3015@gmail.com; 3 Department of Physical Therapy, University of Dayton, 300 College Park, Dayton, OH 45469, USA; jaszekj1@udayton.edu (J.J.); djukicc1@udayton.edu (C.D.); annalbrown24@gmail.com (A.B.); kjackson1@udayton.edu (K.J.)

**Keywords:** continuous human activity monitoring, millimeter-wave radar sensor, PointNet, artificial intelligence, telemedicine, fall alert

## Abstract

Telemedicine has the potential to improve access and delivery of healthcare to diverse and aging populations. Recent advances in technology allow for remote monitoring of physiological measures such as heart rate, oxygen saturation, blood glucose, and blood pressure. However, the ability to accurately detect falls and monitor physical activity remotely without invading privacy or remembering to wear a costly device remains an ongoing concern. Our proposed system utilizes a millimeter-wave (mmwave) radar sensor (IWR6843ISK-ODS) connected to an NVIDIA Jetson Nano board for continuous monitoring of human activity. We developed a PointNet neural network for real-time human activity monitoring that can provide activity data reports, tracking maps, and fall alerts. Using radar helps to safeguard patients’ privacy by abstaining from recording camera images. We evaluated our system for real-time operation and achieved an inference accuracy of 99.5% when recognizing five types of activities: standing, walking, sitting, lying, and falling. Our system would facilitate the ability to detect falls and monitor physical activity in home and institutional settings to improve telemedicine by providing objective data for more timely and targeted interventions. This work demonstrates the potential of artificial intelligence algorithms and mmwave sensors for HAR.

## 1. Introduction

Demands on the healthcare system associated with an aging population pose a significant challenge to nations across the world. Addressing these issues will require the ongoing adaptation of healthcare and social systems [1]. According to the United States Department of Health and Human Services, those aged 65 and older comprised 17% of the population in 2020, but this proportion is projected to rise to 22% by 2040 [2]. Further, the projected increase in the population of those aged 85 and above is anticipated to increase by twofold. Older adults are more prone to chronic and degenerative diseases such as Alzheimer’s, respiratory diseases, diabetes, cardiovascular disease, osteoarthritis, stroke, and other chronic ailments [3] which require frequent medical care, monitoring, and follow-up. Further, many seniors choose to live independently and are often alone for extended periods of time. For example, in 2021, over 27% (15.2 million) of older adults residing in the community lived alone [2]. One major problem for older adults who choose to live alone is their vulnerability to accidental falls, which are experienced by over a quarter of those aged 65 and older annually, leading to three million emergency visits [4]. Recent studies confirm that preventive measures through active monitoring could help curtail these incidents [5].

Clinicians who treat patients with chronic neurological conditions such as stroke, Parkinson’s disease, and multiple sclerosis also encounter challenges in providing effective care. This can be due to difficulty in monitoring and measuring changes in function and activity levels over time and assessing patient compliance with treatment outside of scheduled office visits [6]. Therefore, it would be beneficial if there were accurate and effective ways to continuously monitor patient activity over extended periods of time without infringing on patient privacy. For these reasons, telemedicine and continuous human activity monitoring have become increasingly important components of today’s healthcare system because they can allow clinicians to engage remotely using objective data [7,8].

Telemedicine systems allow for the transmission of patient data from home to healthcare providers, enabling data analysis, diagnosis, and treatment planning [9,10]. Given the scenario that many older people prefer to live independently in their homes, incorporating and improving telemedicine services has become crucial for many healthcare organizations [11], a sentiment supported by the 37% of the population that utilized telemedicine services in 2021 [12]. Telemedicine monitoring facilitates the collection of long-term data, provides analysis reports to healthcare professionals, and enables them to discern both positive and negative trends and patterns in patient behavior. These data are also essential for real-time patient safety monitoring, alerting caregivers and emergency services during incidents such as a fall [13]. This capability is valuable for assessing patient adherence and responses to medical and rehabilitation interventions [14]. Various technologies have been developed for human activity recognition (HAR) and fall detection [15]. However, non-contact mmwave-based radar technology has garnered considerable attention in recent years due to its numerous advantages [16], such as its portability, low cost, and ability to operate in different ambient and temperature conditions. Furthermore, it provides more privacy compared to traditional cameras and is more convenient than wearable devices [17,18].

The integration of mmwave-based radar systems in healthcare signifies notable progress, specifically in improving the availability of high-quality medical care for patients in distant areas, thus narrowing the disparity between healthcare services in rural and urban regions. This technological transition allows healthcare facilities to allocate resources more efficiently to situations that are of higher importance, therefore reducing the difficulties associated with repeated hospital visits for patients with chronic illnesses. Moreover, these advancements enhance in-home nursing services for the elderly and disabled communities, encouraging compliance with therapeutic treatments and improving the distribution of healthcare resources. Crucially, these sophisticated monitoring systems not only enhance the quality and effectiveness of treatment but also lead to significant cost reductions. These advancements play a crucial role in helping healthcare systems effectively address the changing requirements of an aging population, representing a significant advancement in modern healthcare delivery.

While mmwave-based radar technology offers significant advantages for HAR and fall detection, the complexity of the data it generates presents a formidable challenge [19]. Typically, radar signals are composed of high-dimensional point cloud data that is inherently information-rich, requiring advanced processing techniques to extract meaningful insights. Charles et al. [20] recently proposed PointNet, a deep learning architecture that enables the direct classification of point cloud data from mmwave-based radar signals. Their model preserves spatial information by processing point clouds in their original form. The combination of mmwave radar and PointNet can help HAR applications by improving their performance in terms of precision, responsiveness, and versatility across a wide range of scenarios [21]. Accordingly, we utilized the PointNet algorithm to process mmwave radar’s point cloud data for our proposed HAR application to overcome the aforementioned technical limitations. The primary contributions of our work are described below:

- **HAR System:** We present an approach for HAR using the TI mmwave radar sensor in conjunction with PointNet neural networks implemented on the NVIDIA Jetson Nano Graphical Processing Unit (GPU) system. This system offers a non-intrusive and privacy-preserving method for monitoring human activities without using camera imagery. Furthermore, it directly uses point cloud data without additional pre-processing.
- **Real-Time Classification of Common Activities:** Our system achieves real-time monitoring and classification of five common activities, including standing, walking, sitting, lying, and falling, with an accuracy of 99.5%.
- **Comprehensive Activity Analysis:** We provide a novel comprehensive analysis of activities over time and spatial positions, offering valuable insights into human behavior. Our solution includes the ability to generate detailed reports that depict the temporal distribution of each activity and spatial features through tracking maps, ensuring a detailed understanding of human movement patterns.
- **Fall Detection and Alert Mechanism:** Our system includes an alert mechanism that leverages the Twilio Application Programming Interface (API) protocol. This feature allows for prompt notification in the event of a fall, enabling rapid intervention and potentially saving lives.

The ensuing portions of this study are structured as follows: Section 2 of this paper is dedicated to a comprehensive review of prior research on the various methodologies employed for conducting HAR. In Section 3, a detailed description of the system architecture of the mmwave-based HAR is presented. A description of the data preparation and collection, along with the evaluation of the methodology’s effectiveness, can be found in Section 4. In Section 5, the study’s findings and analyses are presented. The limitations and future directions of the study are discussed in Section 6, and the conclusions are outlined in Section 7.

## 2. Human Activity Recognition Approaches and Related Work

Human activity involves a series of actions carried out by one or more individuals to perform an action or task, such as sitting, lying, walking, standing, and falling [22]. The field of HAR has made remarkable advancements over the past decade. The primary objective of HAR is to discern a user’s behavior, enabling computing systems to accurately classify and measure human activity [23].

Today, smart homes are being constructed with HAR to aid the health of the elderly, disabled, and children by continuously monitoring their daily behavior [24]. HAR may be useful for observing daily routines, evaluating health conditions, and assisting elderly or disabled individuals. HAR plays a role in automatic health tracking, enhancements in diagnostic methods and care, and enables remote monitoring in home and institutional settings, thereby improving safety and well-being [25].

**Figure 1:**
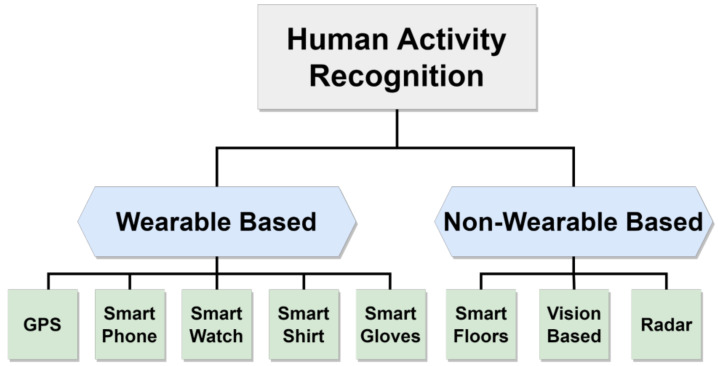
Classification of human activity recognition approaches.

Existing literature in this area often categorizes research based on the features of the devices used, distinguishing between wearable and non-wearable devices, as depicted in Figure 1. Wearable devices encompass smartphones, smartwatches, and smart gloves [26], all capable of tracking human movements. In contrast, non-wearable devices comprise various tools like visual-based systems, intelligent flooring, and radar systems. An illustrative summary of these methodologies is presented in this section, offering a snapshot of the investigations undertaken and a brief overview of diverse applications utilizing these techniques.

Wearable technology has become increasingly useful in capturing detailed data on an individual’s movements and activity patterns through the utilization of sensors placed on the body [15]. This technology includes various devices such as Global Positioning System (GPS) devices, smartwatches, smartphones, smart shirts, and smart gloves. Its application has made notable contributions to the domains of HAR and human–computer interfaces (HCIs) [26]. Nevertheless, it is important to acknowledge that every type of device presents its own set of advantages and disadvantages. For instance, GPS-based systems face obstacles when it comes to accurately identifying specific human poses, and experience signal loss in indoor environments [27]. Smartwatches and smartphones can provide real-time tracking to monitor physical activity and location. They feature monitoring applications that possess the ability to identify health fluctuations and possibly life-threatening occurrences [28,29]. However, smartwatches have disadvantages such as limited battery life, and users must remember to wear them continuously [30]. Further, smartphones encounter issues with sensor inaccuracy when they are kept in pockets or purses [31] and they encounter difficulties in monitoring functions that require direct contact with the body. Other wearable devices, such as the Hexoskin smart shirt [32] and smart textile gloves developed by Itex [33], present alternative options for HAR. However, the persistent need to wear these devices imposes limitations on their utilization in a variety of situations such as when individuals need to take a shower or during sleep [34]. As mentioned before, especially when monitoring older adults, failure to constantly wear monitoring devices can lead to missing unexpected events such as falls [35].

Non-wearable approaches for HAR utilize ambient sensors like camera-based devices, smart floors, and radar systems. Vision-based systems have shown promise in classifying human poses and detecting falls, leveraging advanced computer vision algorithms and high-quality optical sensors [36]. However, challenges like data storage needs, processing complexity, ambient light sensitivity, and privacy concerns hinder their general acceptance [37]. Intelligent floor systems such as carpets and floor tiles provide alternative means for monitoring human movement and posture [38]. A study on a carpet system displayed its ability to use surface force information for 3D human pose analysis but revealed limitations in detecting certain body positions and differentiating similar movements [39]. Recently, radar-based HAR has gained interest due to its ease of deployment in diverse environments, insensitivity to ambient lighting conditions, and maintaining user privacy [18,40].

Mmwave is a subset of radar technology [41], that is relatively low cost, has a compact form factor, and has high-resolution detection capabilities [42]. Further, it can penetrate thin layers of some materials such as fabrics, allowing seamless indoor placement in complex living environments [43]. Commercially available mmwave devices have the capability to create detailed 3D point cloud models of objects. The collected data can be effectively analyzed using edge Artificial Intelligence (AI) algorithms to accurately recreate human movements for HAR applications [44].

The mmwave radar generates point clouds by emitting electromagnetic waves and capturing their reflections as they interact with the object or person. These point clouds represent the spatial distribution of objects and movements, which are then processed to decipher human activities. However, the fluctuating count of cloud points in each frame from mmwave radar introduces challenges in crafting precise activity classifiers, as these typically require fixed input dimensions and order [35]. To address this, researchers commonly standardize the data into forms like micro-Doppler signatures [45,46], image sequences [47,48,49], or 3D voxel grids [19,50] before employing machine learning. This standardization often results in the loss of spatial features [51] and can cause data bloat and related challenges [20].

The proposed approach uses the PointNet network to overcome constraints faced by directly processing raw point cloud data, thereby retaining fine-grained spatial relationships essential for object tracking [52]. As shown in Table 1, our proposed system achieved novel high accuracy compared with prior studies and extracted accurate tracking maps using spatial features. PointNet’s architecture, leveraging shared Multi-Layer Perceptron (MLP), is computationally efficient and lightweight, making it well-suited for real-time HAR applications [20].

## 3. System Overview

This section elucidates the primary components of our proposed system for continuous HAR using a mmwave radar sensor. The system encompasses a mmwave radar sensor for monitoring and an NVIDIA Jetson Nano GPU board to accurately discern five distinct activities: standing, walking, sitting, lying, and falling, utilizing the PointNet deep learning algorithms. Additionally, our proposed system uses an alert feature for care providers, designed to notify them of fall events via Hypertext Transfer Protocol (HTTP) requests via Twilio, which sends SMS notifications and initiates alert calls.

### 3.1. Millimeter-Wave Radar Sensor (IWR6843ISK-ODS)

Texas Instruments’ radar sensors employ frequency-modulated continuous wave (FMCW) to determine the range, velocity, and angle of objects through frequency-modulated signals [41]. The shorter wavelength of mmwave radars, falling within the millimeter range, enhances accuracy and enables 3D visualization using point clouds, accurately identifying human postures [13]. We use the Texas Instruments (TI) 60 GHz IWR6843ISK-ODS mmwave radar for real-time point cloud generation in 3D Cartesian coordinates, along with velocity information, to track individuals within its field of view (FoV) [60].

**Figure 2:**
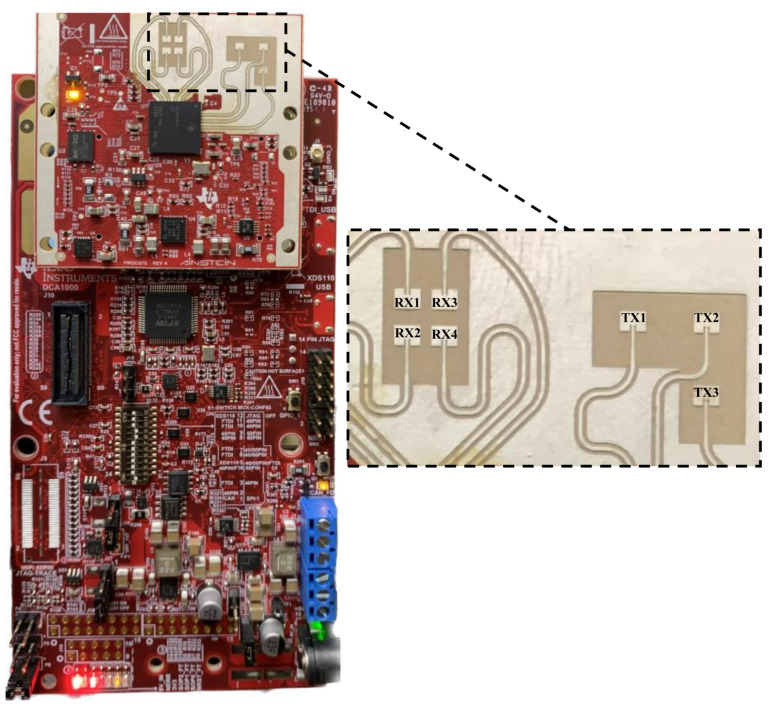
Texas Instruments IWR6843ISK-ODS mmwave sensor with MMWAVEICBOOST.

The IWR6843ISK-ODS mmwave sensor features a short-range antenna with a broad FoV, interfacing with the MMWAVEICBOOST carrier card. Its evaluation module houses a transceiver paired with an antenna, facilitating point cloud data access via USB, as depicted in Figure 2. The key metrics of IWR6843ISK-ODS are listed in Table 2 [61]. The sensor, with four receivers and three transmitters, can detect individuals up to 18 m away. The maximum detectable range for a human is determined using the link budget formula. This formula relies on factors like detection SNR, radar cross-section, radar device RF performance, antenna gains, and chirp parameters, which are calculated by:

(1) $$r_{max,d}=\sqrt[{4}]{\frac{\sigma P_{T_{x}}G_{T_{x}}G_{R_{x}}\lambda^{2}T_{c}N_{c}N_{T_{x}}N_{R_{x}}}{{\left(4\pi \right)}^{3}KT_{e}L\eta SNR}}$$

where λ is the wavelength, *K* is Boltzmann’s constant, $T_{e}$ Ambient temperature, *L* is total system loss. The IWR6843ISK-ODS sensor has built-in detection and tracking algorithms to ascertain individual locations, monitor movements, and track all moving objects in the scene, even if they are seated or lying down.

**Figure 3:**
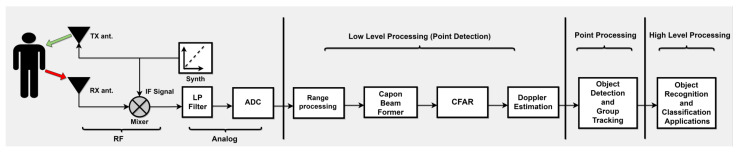
Mmwave signal processing chain elements.

The detection process commences with a synthesizer emitting a chirp, which is transmitted by the transmit antenna $T_{x}$ (2) and then reflected off objects back chirp reflected at the receive antenna $R_{x}$ (3), as illustrated in Figure 3.

(2) $$T_{x=sin\left[w_{1}t+\Phi_{1}\right]}$$

(3) $$R_{x=sin\left[w_{2}t+\Phi_{2}\right]}$$

The IF signal, as depicted in Equation (1), is a sinusoidal waveform whose instantaneous frequency and phase are determined by the disparities in the instantaneous frequency and phase of the input sinusoidal signals. These signals are combined to create an intermediate frequency (IF) signal (4), which is subsequently digitized for further analysis.

(4) $$IF=T_{x}+R_{x}=sin\left[\left(w_{1}-w_{2}\right)t+\left(\Phi_{1}-\Phi_{2}\right)\right]$$

This creates measurement vectors or point clouds that show the physical properties of the scene [41,61]. Obtaining raw 3D radar data is the first step in processing radar signals. Each antenna then goes through range processing using 1D windowing and 1D Fast Fourier Transform (FFT). Following this, a static clutter removal procedure is employed to filter out stationary objects, isolating signals emanating from moving objects. Techniques such as capon beamforming are utilized to formulate a range-azimuth heatmap, with object identification being executed through a constant false alarm rate approach. Further refinement is accomplished through elevation and Doppler estimations, which ascertain the angular positions and radial velocities of detected objects [61].

Transitioning to the tracking phase, the focus shifts toward identifying and tracking clusters within the point cloud. The tracking layer leverages the point cloud data to pinpoint and track these clusters, culminating in a target list. Each point encapsulates values such as range, azimuth angle, and radial velocity. Through this layer, targets are identified and a list is compiled, encapsulating attributes like track ID, position, velocity, and size, which prove instrumental in subsequent tasks like visualization and object categorization [62]. Exploiting the radars’ expansive bandwidth and 8 cm range resolution, multiple target points are derived from the reflections off the human body, with point clouds in each frame representing these targets. The mmwave radar sensor’s point clouds each contain 3D coordinates and velocity, among other characteristics. The term “frame” denotes the data set captured by the radar at each instance. Data points within each frame correspond to target movement, rendering them pivotal for precise target location and suitable for classification and recognition actions in high-level processing.

### 3.2. NVIDIA Jetson Nano GPU

The NVIDIA Jetson Nano is a compact integrated system-on-module (SoM) and development package adept at executing multiple neural networks simultaneously. This single-board computer (SBC) balances the computational capability essential for modern AI applications with its small size, low cost, and low energy consumption while operating under a power requirement of less than 5 W. It facilitates the deployment of AI frameworks for tasks like image categorization, object detection, segmentation, and audio processing [63]. The characteristics of the NVIDIA Jetson Nano system are summarized in Table 3 [64].

Additionally, NVIDIA offers the TensorRT toolkit to enhance the effectiveness of deep learning layers on Jetson devices. TensorRT is a high-quality deep learning inference software development kit (SDK) that melds an inference optimizer with runtime for low latency and robust throughput. Compatible with training frameworks like TensorFlow and PyTorch, it efficiently executes pre-trained networks on NVIDIA’s hardware. Compared to standard GPU-based inference, TensorRT notably enhances performance and reduces power consumption [65,66].

### 3.3. PointNet Neural Network

**Figure 4:**
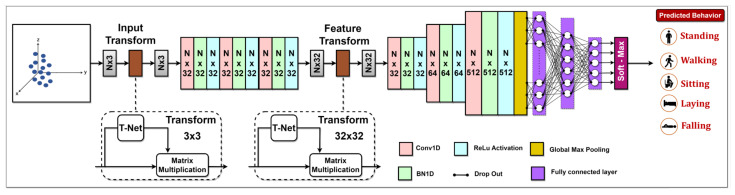
PointNet Model Architecture.

In our methodology, the PointNet architecture processes raw point cloud data to classify the points into one of five categories: standing, walking, sitting, lying, or falling. The architecture shown in Figure 4 begins with an input layer for a set of cloud points $p=\left(p_{1},p_{2},\cdots ,p_{n}\right)$, where *n* represents the total number point count, with each point being characterized by its Cartesian coordinates. $p_{i}=\left(x,y,z\right)$ in a 3D Euclidean space, designed to accommodate point clouds with a shape representing the number of points and three coordinates. Following the input layer, the model proceeds with an input transformation network (T-net), which operates on the raw point cloud data. A significant aspect of PointNet is the incorporation of T-net, which aims to spatially align the point cloud data, helping in learning rotation and translation-invariant features. In the architecture of the PointNet model, following the initial T-net, there are three 1D convolutional layers (Conv1D), each comprising 32 filters. Following each convolution operation, the convolution output undergoes 1D batch normalization (BN1D) for normalization purposes. Subsequently, a Rectified Linear Unit (ReLU) activation function is employed to introduce nonlinearity, aiding the model in learning from the data. This is achieved by applying $ReLU\left(y_{i}\right)=max\left(0,y_{i}\right)$. The model then goes through another T-net to align the feature representations. It also aligns the feature space with a transformation matrix, using a regularization term to ensure near orthogonality and stable optimization.

(5) $$T_{reg}={∥I-KK^{T}∥}_{F}^{2}$$

where *I* is the identity matrix, *K* is the feature alignment matrix predicted by the T-net, and *F* denotes the Frobenius norm. This alignment is crucial as it improves the model’s ability to generalize across varied spatial orientations of data. Then another Conv1D with 32 filters, one with 64 filters, and a final one with a whopping 512 filters for deeper feature extraction. After convolutional layers, a global max pooling layer condenses the feature maps into a single global feature vector. Following the pooling layer, the global feature vector is passed through a series of fully connected layers known as the MLP. The MLP comprises three layers. The first two layers have 256 and 128 units, respectively. 1D batch normalization and ReLU activation follow each of these layers, ensuring a normalized and non-linear transformation of the data. Additionally, dropout layers with a rate of 0.3 are interspersed between these fully connected layers for regularization to reduce the risk of overfitting during training. The third dense layer is designed to reshape the features for subsequent operations. The MLP serves to further process the extracted features, making them suitable for the final classification stage. The network ends with a fully connected layer that has a set number of class units and a SoftMax activation function that sorts the input point cloud into one of five classes. The entire process enables PointNet to derive human posture classifications from 3D data and utilize these to create spatial feature tracking maps. The finalized model is then stored to be used on the Nvidia Jetson Nano for real-time execution.

### 3.4. Twilio API Programmable Protocol Messages

**Figure 5:**
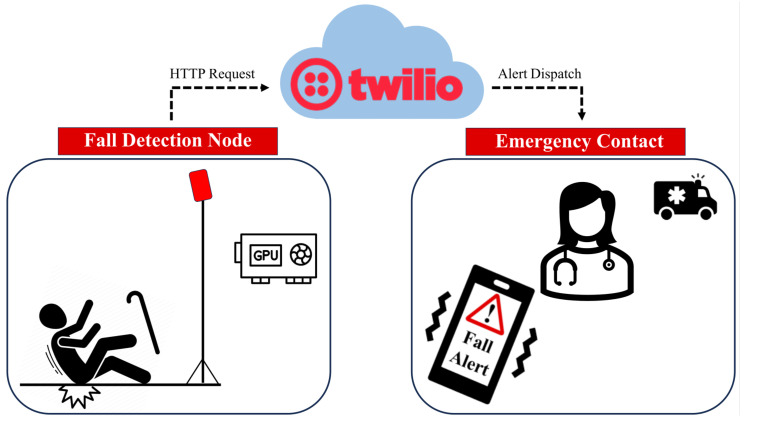
Falling Alert System Architecture.

Text messages are an efficient means of delivering timely alerts, particularly within the healthcare sector, in the case of critical events like falls. Therefore, we use the Twilio Application Service to make a fall alert SMS system. Twilio is a web service API that offers programmable communication capabilities for the transmission and receiving of text messages, phone calls, and several other modes of communication. The HTTP protocol is used to transmit administrator notifications to Twilio REST APIs, which allow developers to send SMSs and calls, as shown in Figure 5. Twilio SIM cards, administered via APIs and the Twilio Console, provide tailored solutions for IoT applications [67]. Therefore, it enables effective communication, rapid event response, and improved alerting systems. This feature is crucial in facilitating patient communication and notifying care providers about fall incidents.

As illustrated in Figure 5, upon the detection of a fall, the system swiftly initiates an HTTP request to the Twilio cloud platform. Upon receipt of this request, the server evaluates the information and leverages the Twilio REST API to instruct Twilio to dispatch a predetermined SMS bearing the critical fall alert message. Simultaneously, a voice call is initiated by the platform, alerting the designated emergency contact through an automated voice message about the detected fall. This contact could be a healthcare practitioner or a close relative. The entire process commences with fall detection on our Jetson Nano model, then moves to the Twilio cloud platform, where it interfaces with the Twilio service, and culminates in notifying the selected emergency contact through both an SMS and voice call alert.

## 4. Experiment Setup and Data Collection

### 4.1. Experimental Setup

**Figure 6:**
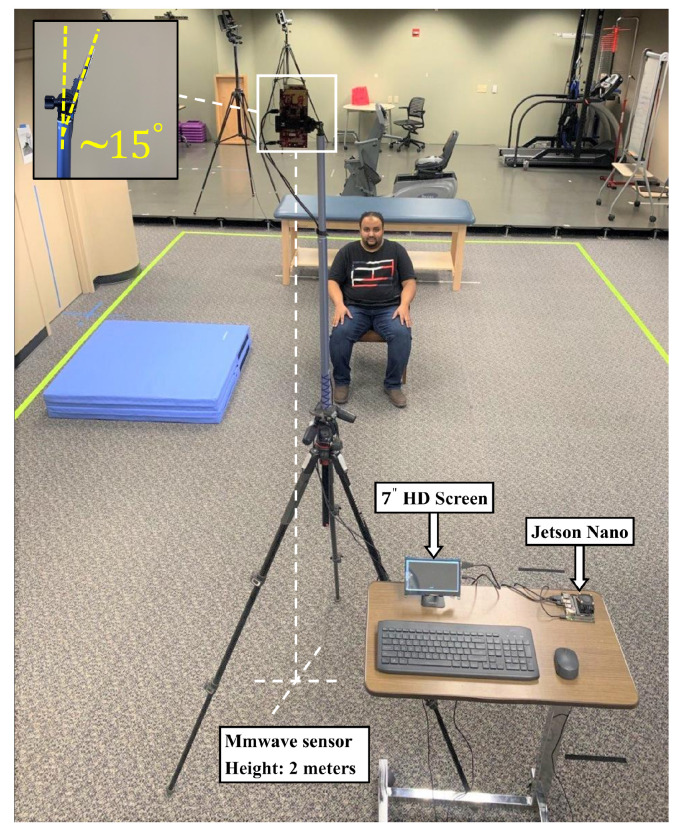
The experimental setup.

The experimental setup aimed to acquire data on human activities within a simulated home environment. A mmwave radar sensor, specifically the IWR6843ISK-ODS model, was positioned on tripods at a height of 2 m, as shown in Figure 6. The radar was tilted by 15 degrees in the depth-elevation plane to enhance coverage over a designated 12-square-meter area. This area was designed to mimic a living room setting, with furniture like chairs, floor mats, beds, and walking space. The primary target for detection was the human subject, as depicted in Figure 6.

### 4.2. Data Collection

Our study received permission from the University of Dayton Institutional Review Board (IRB) for data collection involving a cohort of 89 healthy adults in accordance with ethical guidelines. The data collection encompassed 57 male participants, while 32 were female. Taking into account a broad spectrum of height and weight variations is necessary to generate training data that can represent a diverse population, which leads to better generalization and reduces biases. A summary of the participant’s demographic characteristics can be found in Table 4. Further, incorporating a diversified set of weight and height parameters during the training phase is crucial to ensuring that the model encompasses an ample quantity of varied and representative data points. This allows the model to effectively generalize its classification capabilities when applied to the test data.

**Figure 7:**
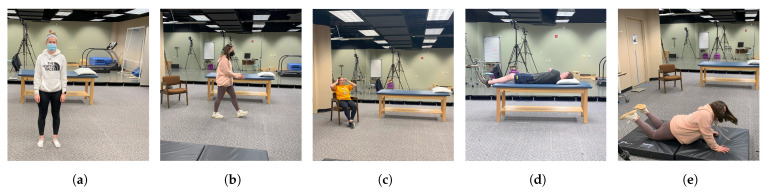
Samples of who participates in the collecting data phase. (**a**) Standing. (**b**) Walking. (**c**) Sitting. (**d**) Lying. (**e**) Falling.

Our study centered on the recognition of five distinct bodily positions: standing, walking, sitting, lying, and falling, which constitute the five output categories, as depicted in Figure 7. The process of radar signal handling, detailed in Section 3, commences with the emission of chirp signals by the radar’s transmitters. Subsequently, the receiver captures these signals following their interaction with the participants, leading to the extraction of data pertaining to trackable objects. Also, our dataset is carefully labeled to differentiate five activities, reducing overlap and enhancing clarity. This dataset comprises key attributes, including track ID, position, velocity, and physical dimensions. Throughout the study protocol, each participant engaged in a sequence of activities. These activities encompassed standing before the sensor for a duration of 30 s, engaging in random walking movements within the sensor’s coverage area for an additional 30 s, assuming a seated posture on a chair for 30 s, lying on a bed for 30 s with intermittent rolling to both sides, and ultimately transitioning from a standing position to an abrupt fall, remaining in the fallen position for an additional 30 s. The mmwave radar sensor effectively captured the participants’ movements and generated point cloud data. This collected data file for each participant’s activity contains numerous data frames, which significantly increases the dataset’s size. Data collection was conducted across various positions within the room to ensure diversity and enhance data quality. Five samples, each spanning a duration of 30 s, were acquired from each participant during this process.

### 4.3. Data Analysis

To evaluate the efficacy of our PointNet model and its architecture, a comprehensive array of evaluation metrics was used. These encompassed fundamental measures such as the Receiver Operating Curve (ROC), F1-score, precision, recall, and the utilization of confusion matrixes.

The data were split into training (80%) and validation (20%) sets to adjust hyperparameters, prevent overfitting, and evaluate the model’s performance on unseen data. The model was trained with the training dataset, allowing its performance to be evaluated and compared to the validation dataset, which was not used during training.

### 4.4. Receiver Operating Characteristic (ROC) Curve

The assessment of the model included ROC curve analysis, which is commonly utilized as a diagnostic instrument for evaluating the performance of classification models. This is achieved by evaluating the trade-off between the true positive rate and the false positive rate across different discriminatory thresholds. The present study involved the application of ROC analysis to assess the accuracy of the radar in identifying the five predetermined physical activities.

**Figure 8:**
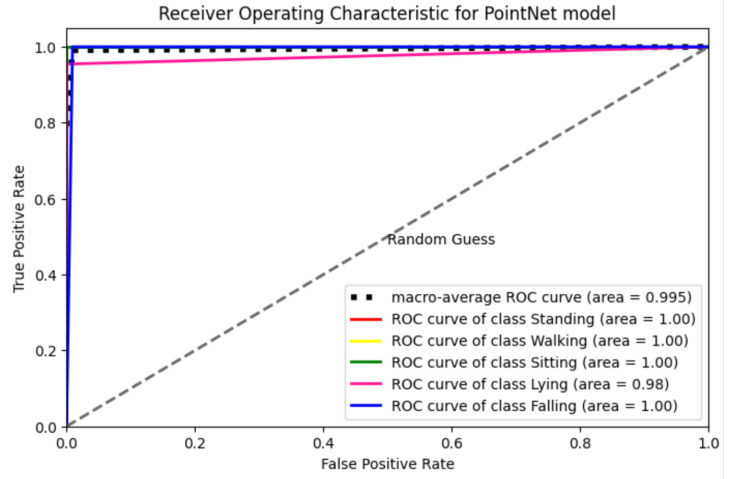
The ROC Curve of the PointNet model.

The findings depicted in Figure 8 highlight the performance of the proposed model across the activity categories. The ROC curves for these classes not only demonstrate prominent peaks but also validate the model’s performance in accurately distinguishing various activities, including scenarios involving fall detection. The ROC curves for all five classes exhibit similar and parallel trajectories, indicating a stable and equally proficient true recognition rate across various positions. The mean ROC curve suggests that the efficacy of the HAR utilizing mmwave radar for the classification of five distinct activities is 99.5%, highlighting the effectiveness of the proposed system in accurately discriminating between various tasks.

### 4.5. Confusion Matrixes

**Figure 9:**
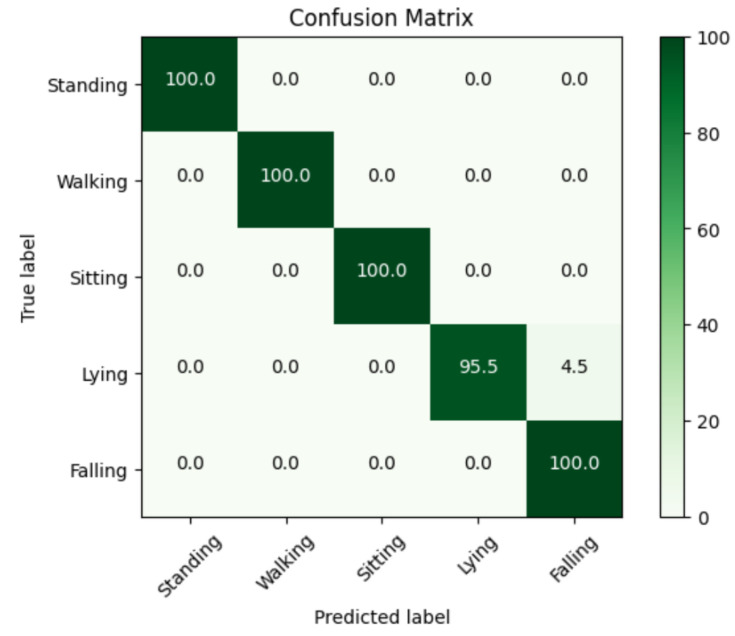
The Confusion Matrix of the PointNet model.

The confusion matrix is a useful tool for assessing the effectiveness of classification models. The abscissa represents the true labels, whereas the ordinate represents the predicted labels. Enhanced model performance is shown by a higher concentration of predicted values along the diagonal of the confusion matrix. In Figure 9, the confusion matrix visually portrays the classification performance. Notably, all activity classes are classified with excellent accuracy. While ‘Lying’ and ‘Falling’ have certain similarities in terms of their proximity to the ground and body posture, the key distinguishing feature between them is the height at which the human target is positioned above the ground surface, which differs greatly between the two. Radar’s ability to generate a three-dimensional representation allows it to overcome these hurdles, resulting in highly effective classification performance.

### 4.6. F1-Score, Precision, Recall

An evaluation of the classification performance is conducted by calculating metrics such as “F1-score”, “Precision”, and “ Recall”. These measures consider the quantities of true positives (TPs), true negatives (TNs), false positives (FPs), and false negatives (FNs). Mathematically, the metrics are defined in the following manner:

The F1-score is a metric that calculates a weighted average of precision and recall.

(6) $$F1\text{-}score=2\times \frac{Recall\times Precision}{Recall+Precision}$$

The precision metric is formally defined as the ratio produced by dividing the count of true positive instances by the sum of the count of true positive instances and the count of false positive instances.

(7) $$Precision=\frac{TPs}{TPs+FPs}$$

The mathematical expression for the recall formula involves dividing the number of true positives by the sum of true positives and false negatives.

(8) $$Recall=\frac{TPs}{FNs+TPs}$$

A summary of the Evaluation metric results can be found in Table 5.

## 5. Results and Discussions

This section presents the results of our proposed system for continuous monitoring of human activities. We conducted two separate experiments, one for a short period (5 min) and another for a longer duration (30 min), which provided comprehensive data analysis reports regarding activity distribution over time and space. We also tested the fall detection and alert mechanism, which is connected via the Twilio REST API protocol, for notifying supervisory personnel in the event of a fall.

### 5.1. Real-time Detection of Human Activity

**Figure 10:**
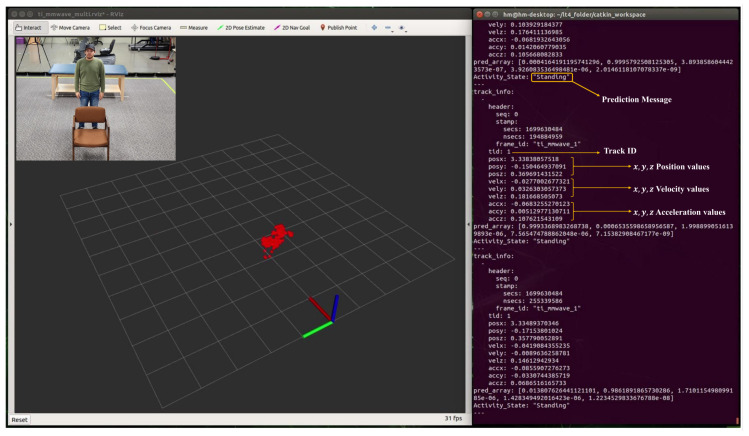
Real-time detection of a person in a standing position.

**Figure 11:**
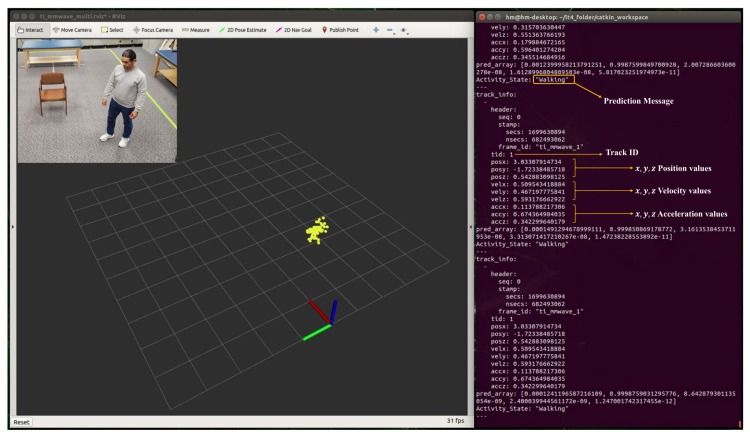
Real-time detection of a person in a walking position.

**Figure 12:**
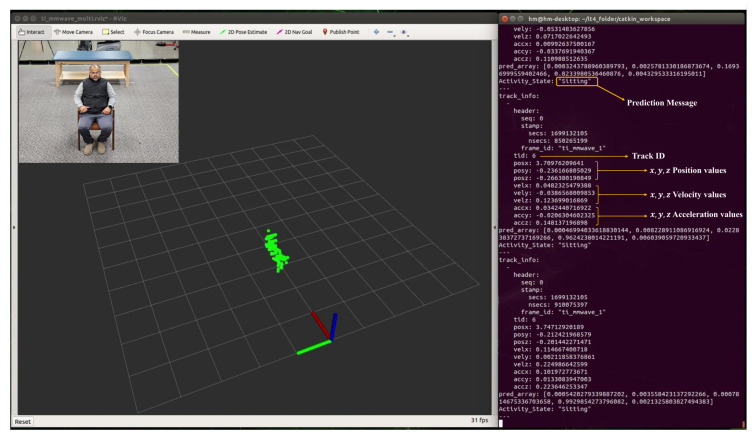
Real-time detection of a person in a sitting position.

**Figure 13:**
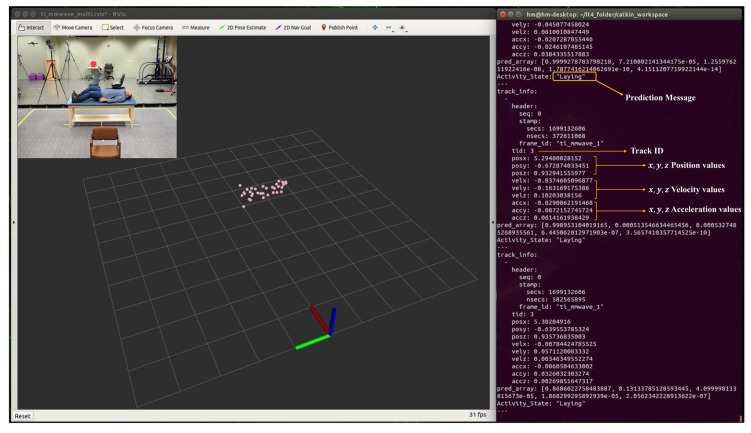
Real-time detection of a person in a lying position.

**Figure 14:**
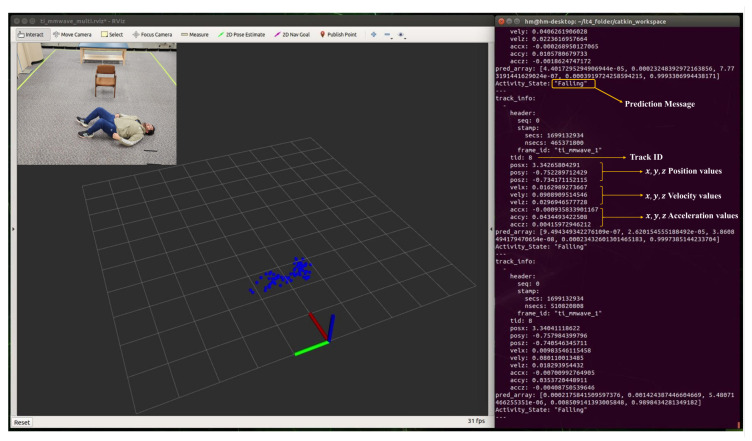
Real-time detection of a person in a falling position.

During real-time monitoring of the five different activities (standing, walking, sitting, lying, and falling), we assigned each activity a specific color: red for standing, yellow for walking, green for sitting, pink for lying, and blue for falling, which gives an easy way to discern between each. This is shown in the following Figure 10, Figure 11, Figure 12, Figure 13 and Figure 14, where each human target appears in the field of view by the mmwave sensor and is given a specific trackID, and then the cloud points are recolored according to the current activity and the prediction message that is displayed on the Rviz screen of the Jetson Nano in the “Activity_State” parameter with position, velocity, and acceleration values of *x*, *y*, and *z* dimensions. These values are continuously updated to provide a comprehensive description of the target’s spatial location in a Cartesian coordinate system. Real images have been added to the figures for illustrative purposes.

### 5.2. Short-Term Monitoring of Human Activity

**Figure 15:**
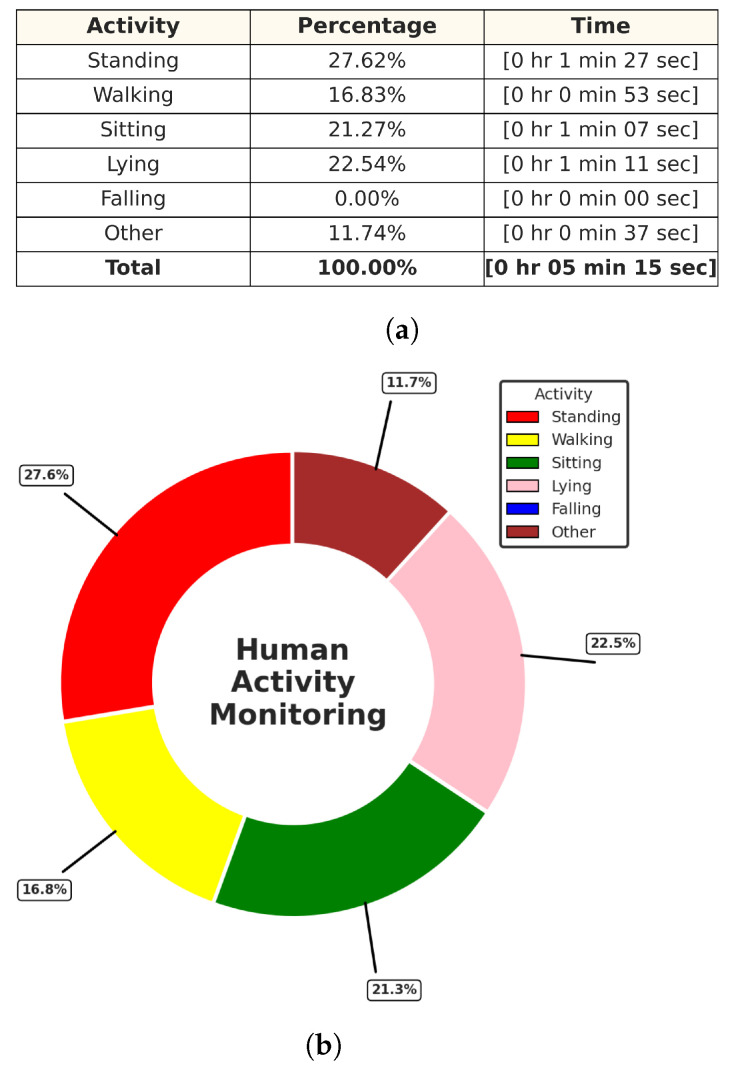
Activities monitoring report of track ID: 1. (**a**) Table of the time distribution of activities. (**b**) Pie chart of the time distribution of activities.

**Figure 16:**
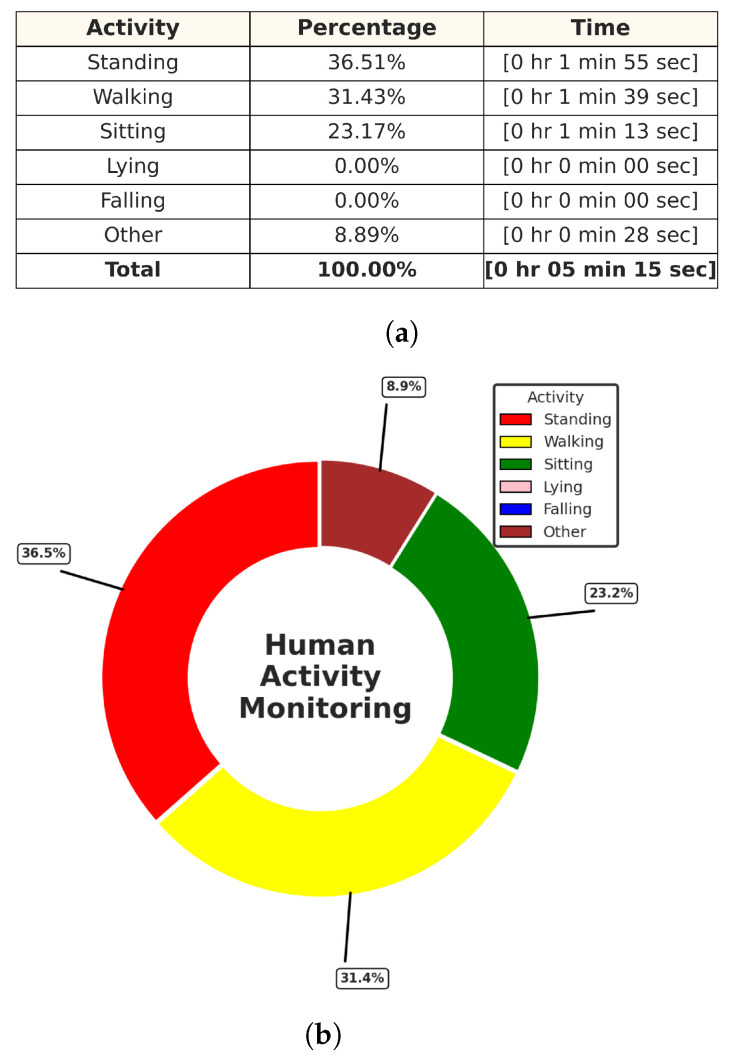
Activities monitoring report of track ID: 2. (**a**) Table of the time distribution of activities. (**b**) Pie chart of the time distribution of activities.

**Figure 17:**
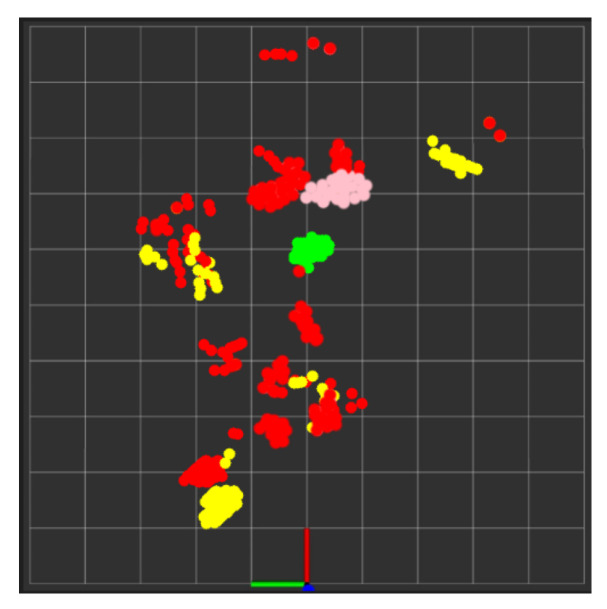
Tracking map of trackID: 1.

**Figure 18:**
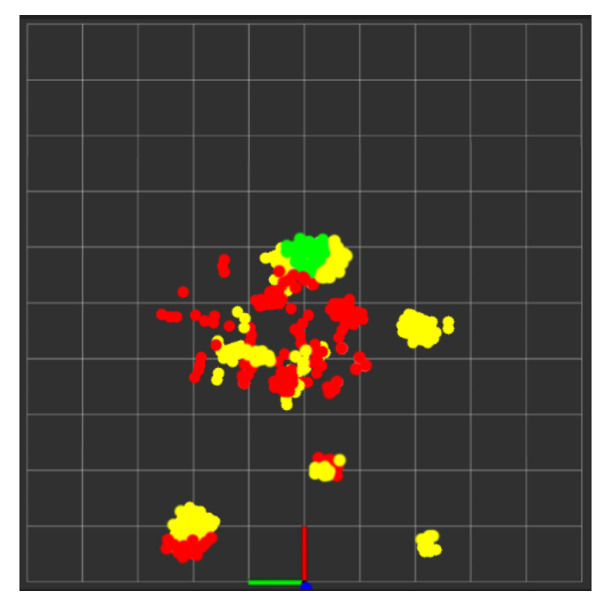
Tracking map of trackID: 2.

In this experiment test, we monitored two human targets for a short period of time (5 min), and then the system produced a separate folder for each target identified by their trackID, which contained the time distribution of each activity with its percentage in a table along with a colored pie chart for quick and easy visualization, as shown in Figure 15 and Figure 16. Additionally, we tested the ability of our PoinetNet algorithm to keep the spatial feature for creating a 2D tracking map that clarifies the position with the corresponding colors assigned for each activity that provides an overview of the monitored room, as shown in Figure 17 and Figure 18, where the axes in the lower center indicate the sensor location in the room. These features can provide healthcare providers with a comprehensive perspective of their patients’ activity levels over time as well as the specific locations of each activity, including falls. This can help healthcare professionals assess whether a fall may be attributable to surrounding environmental factors, as well as know the specific activity that was being performed immediately preceding the fall so that future falls might be prevented.

### 5.3. Long-Term Monitoring of Human Activity

**Figure 19:**
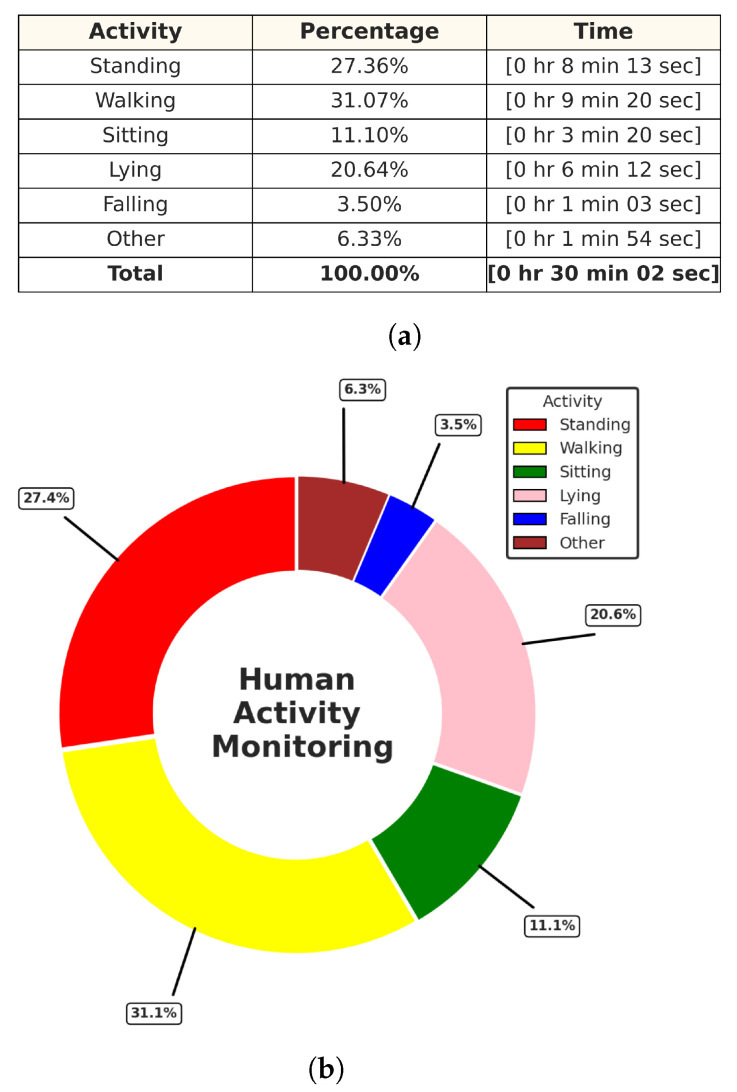
Activities monitoring report of track ID: 3. (**a**) Table of the time distribution of activities. (**b**) Pie chart of the time distribution of activities.

**Figure 20:**
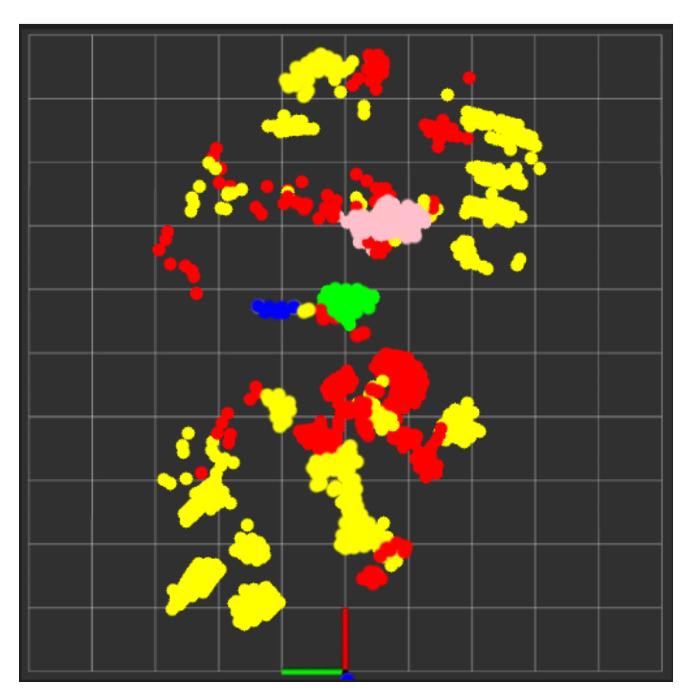
Tracking map of trackID: 3.

**Figure 21:**
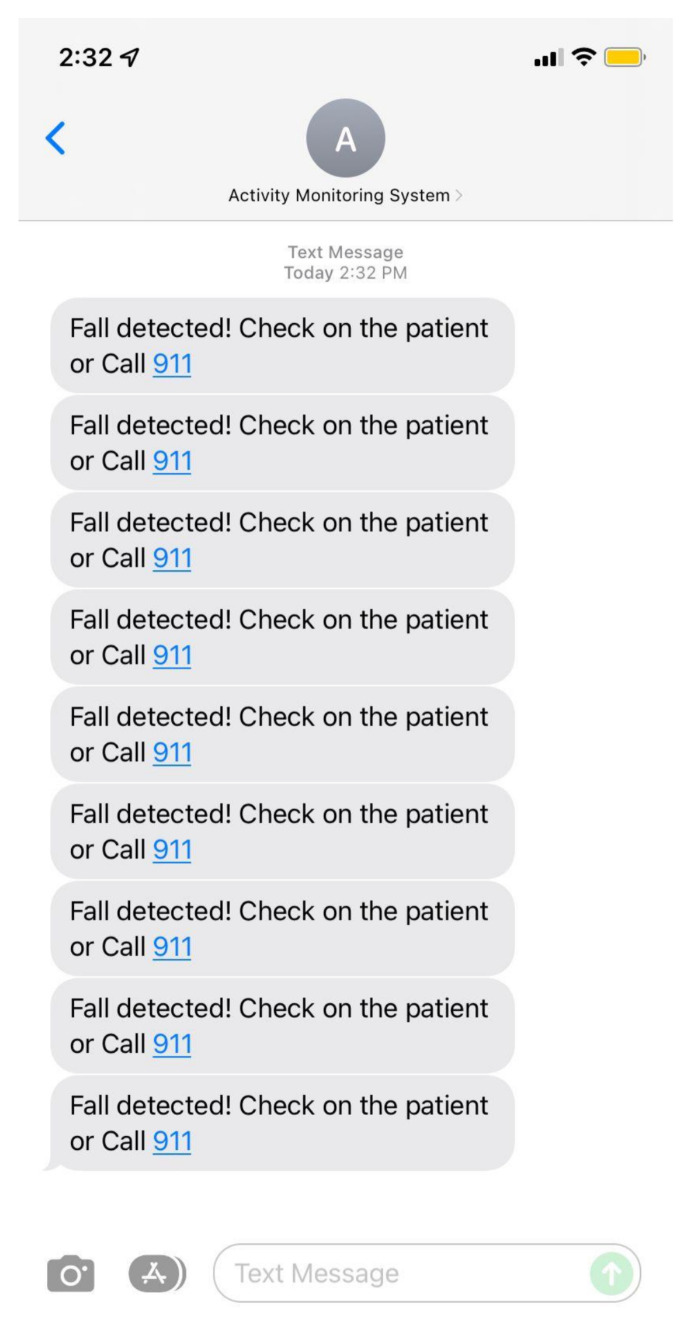
SMS Fall event Alert.

In this experiment, we monitored one human target for about 30 min, and after that, the system produced a separate folder for the target as we did in the previous experiment shown in Figure 19 and Figure 20. Additionally, we tested the fall alert feature to see how accurately a fall was detected by the system. If a fall was detected, the Twilio REST API was immediately activated to send an SMS notification and make an alert call. The resulting SMS notification is shown in Figure 21.

## 6. Limitations and Future Directions

Although our work demonstrates the accuracy and feasibility of mmwave radar for classifying and recording human activity, there are limitations to our findings. One limitation is that we used primarily younger healthy subjects to train the model. Therefore, additional validation in older adults and persons with significant movement impairments such as stroke and Parkinson’s disease who also use assistive devices is warranted. Another limitation of this study was that our testing took place in a simulated living environment with limited obstructions. Additional testing in home and institutional settings with a variety of room layouts and obstructions would be beneficial. Additionally, while mmwave radar can recognize and track more than one person in a room, work remains to improve the ability to identify each individual in the room so that the tracking data can be attributed to the correct individual.

While mmwave radar shows promise as a low-cost and portable solution for HAR, there are still barriers to its widespread implementation. First, suitable Health Insurance Portability and Accountability Act (HIPAA) compliant software and apps will need to be developed that are simple to use and will provide relevant data in a usable format so that healthcare providers can make informed decisions. Integration with smart home systems such as video cameras, voice-activated devices, lighting, etc. would also improve the usefulness of the device but would require additional effort. Work still needs to be performed to determine the optimal coverage area so that the radar can be installed in the correct locations and the number of radar units needed for a home or facility can be easily determined. Lastly, even though radar does not record video images, privacy issues should still be considered carefully as radar systems continue to improve their ability to recognize and record human activity.

## 7. Conclusions

We describe a mmwave radar-based system that can accurately and efficiently classify and monitor five distinct activities: standing, walking, sitting, lying, and falling in real time and over extended periods. The purpose of our work was to demonstrate its use as a tool for telemedicine in home and institutional settings so that caregivers and healthcare providers can engage in remote activity monitoring. The proposed system was developed on an NVIDIA Jetson Nano platform that uses PointNet neural networks to manage the cloud point data from the mmwave radar system. Our methodology does not depend on intermediary representations, such as 3D voxels or pictures, and maintains spatial linkages that are essential for object tracking. As a result, the proposed system demonstrates the capacity to accurately identify and classify five distinct activities in real time, regardless of whether they involve a single target or several targets, with 99.5% accuracy. Our proposed system offers detailed analyses and reports of activities over time and space, providing insights into human behavior. It can generate reports showing the time period of activities and spatial tracking maps for a more comprehensive understanding of movement patterns. Finally, it incorporates functionality for sending a fall alert message and a call to healthcare providers following a fall so that an immediate response can occur.

## Figures and Tables

**Table 1:** Overview on the mmwave radar with machine learning for detecting simple HAR studies.

Ref.	Preprocessing Methods	Sensor Type	Model	Activity Detection	Overall Accuracy
[45]	Micro-Doppler Signatures	TI AWR1642	CNN ^1^	Walking, swinging hands, sitting, and shifting.	95.19%
[46]	Micro-Doppler Signatures	TI AWR1642	CNN	Standing, walking, falling, swing, seizure, restless.	98.7%
[53]	Micro-Doppler Signatures	TI IWR6843	DNN ^2^	Standing, running, jumping jacks, jumping, jogging, squats.	95%
[54]	Micro-Doppler Signatures	TI IWR6843ISK	CNN	Stand, sit, move toward, away, pick up something from ground, left, right, and stay still.	91%
[55]	Micro-Doppler	TI xWR14xx	RNN ^3^	Stand up, sit down, walk, fall, get in, lie down,	NA ^4^
	Signatures	TI xWR68xx		roll in, sit in, and get out of bed.	
[56]	Dual-Micro Motion Signatures	TI AWR1642	CNN	Standing, sitting, walking, running, jumping, punching, bending, and climbing.	98%
[57]	Reflection	Two	LSTM ^5^	Falling, walking, pickup, stand up, boxing, sitting,	80%
	Heatmap	TI IWR1642		and Jogging.	
[58]	Doppler Maps	TI AWR1642	PCA ^6^	Fast walking, slow walking (with swinging hands, or without swinging hands), and limping.	96.1%
[59]	Spatial-Temporal Heatmaps	TI AWR1642	CNN	14 Common in-home full-body workout.	97%
[47]	Heatmap Images	TI IWR1443	CNN	Standing, walking, and sitting.	71%
[48]	Doppler Images	TI AWR1642	SVM ^7^	Stand up, pick up, drink while standing, walk, sit down.	95%
[49]	Doppler Images	TI AWR1642	SVM	Shoulder press, lateral raise, dumbbell, squat, boxing, right and left triceps.	NA
[19]	Voxelization	TI IWR1443	T-D ^8^ CNN	Walking, jumping, jumping jacks, squats and boxing.	90.47%
			B-D ^9^ LSTM		
[50]	Voxelization	TI IWR1443	CNN	Sitting posture with various directions.	99%
[21]	Raw Points Cloud	TI IWR1843	PointNet	Walking, rotating, waving, stooping, and falling.	95.40%
This	Raw Points Cloud	TI IWR6843	PointNet	Standing, walking, sitting, lying, falling.	99.5%
Work					

^1^ CNN: Convolutional Neural Network. ^2^ DNN: Deep Neural Network. ^3^ RNN: Recurrent Neural Network. ^4^ NA: Not Available. ^5^ LSTM: Long Short-Term Memory. ^6^ PCA: principal Component Analysis. ^7^ SVM: Support Vector Machine. ^8^ T-D: Time-distributed. ^9^ B-D: Bi-directional.

**Table 2:** Parameters of the mmwave Radar Sensor IWR6843ISK-ODS.

Parameter	Physical Description
Type	FMCW
Frequency Band	60–64 GHz
Start Frequency (*f_o_*)	60.75 GHz
Idle Time (*T_idle_*)	30 μs
bandwidth (B)	1780.41 MHz
Number of Transmitters (*T_x_*)	3
Number of Receivers *(R_x_*)	4
Total virtual antennas (*N_T_x__, N_R_x__*)	12
Transmit power (*P_T_x__*)	−10 dBm
Noise figure of the receiver (η)	16 dB
Combined *T_x_*/*R_x_* antenna gain (*G_T_x__, G_R_x__*)	16 dB
Azimuth (FoV)	120${}^{\circ }$
Elevation (FoV)	120${}^{\circ }$
Chirp time (*T_c_*)	32.5 μs
Inter-Chirp time (*T_r_*)	267.30 μs
Number of chirps per frame (*N_c_*)	96
Maximum beat frequency (*f_b_*)	2.66 MHz
Center Frequency (*f_c_*)	63.01 GHz
Required detection (SNR)	12 dB
Maximum unambiguous range (*r_max,u_*) ^1^	7.28 m
Maximum detection range based on SNR (*r_max,d_*)	18.27 m
Maximum unambiguous velocity (*v_max_*) ^2^	4.45 m/s
Range resolution (δ*_r_*) ^3^	0.0842 m
Velocity resolution (δ*_v_*) ^4^	0.0928 m/s

^1^*r_max,u_* = $\frac{cf_{b}}{2K}$, ^2^ *v_max_* = $\frac{c}{4T_{r}f_{c}}$, ^3^ δ*_r_* = $\frac{c}{2B}$, ^4^ δ*_v_* = $\frac{c}{N_{c}T_{r}f_{c}}$.

**Table 3:** The NVIDIA Jetson Nano System-on-Module’s characteristics.

Parameter	Physical Description
GPU	128 cores NVIDIA Maxwell architecture
CPU	ARM Cortex-A57 multiprocessor core (Quad-core) unit
RAM	64-bit LPDDR4
Memory Capacity	4 GB
Max Memory Bus Frequency	1600 MHz
Peak Bandwidth	25.6 GB/s
Storage	16 GB, eMMC 5.1
Power	5 Watt
Mechanical	69.6 mm × 45 mm, 260-pin edge connector

**Table 4:** The demographic details of the participants.

Parameter	Mean ± SD (Range)
Age	24 ± 7.42 (21–53)
Height (cm)	169 ± 5.32 (158–186)
weight (kg)	76 ± 11.53 (55–115)
BMI ^1^ (kg/cm^2^)	25.44 ± 4.36 (19.56–40.55)
Gender (M/F) ^2^	57/32

^1^ BMI: Body Mass Index. ^2^ M: Male, F: Female.

**Table 5:** The Evaluation metric of the PointNet model.

	Standing	Walking	Sitting	Lying	Falling
**F1-score**	1.00	1.00	1.00	0.9767	0.9756
**Precision**	1.00	1.00	1.00	1.00	0.9524
**Recall**	1.00	1.00	1.00	0.9545	1.00

## Data Availability

The data presented in this study are available upon request from the A.K.A author.
